# Factors Associated with the Decision to Decline Chemotherapy in Metastatic Non-Small Cell Lung Cancer

**DOI:** 10.3390/cancers15061686

**Published:** 2023-03-09

**Authors:** Iktej Singh Jabbal, Saad Sabbagh, Mira Itani, Barbara Dominguez, Mohamed Mohanna, Valencia Henry, Hong Liang, Diana Saravia, Tiffany George, Zeina Nahleh, Evan Alley, Rafael Arteta-Bulos

**Affiliations:** 1 Department of Hematology/Oncology, Maroone Cancer Center, Cleveland Clinic Florida, Weston, FL 33331, USA; 2 OMS-II, Edward Via College of Osteopathic Medicine-Carolinas Campus, Spartanburg, SC 29303, USA; 3 Department of Clinical Research, Cleveland Clinic Florida, Weston, FL 33331, USA

**Keywords:** lung cancer, refusal of treatment, disparities in cancer care, chemotherapy

## Abstract

**Simple Summary:**

In a cohort of patients diagnosed with metastatic non-small cell lung cancer (NSCLC) where chemotherapy is an integral part of the treatment, shedding light on the characteristics of patients who refuse chemotherapy provides valuable data on the possible reasons and aid in recommending strategies to narrow the gaps in survival outcomes. This original contribution extracted from data in the National Cancer Database (NCDB), one of the largest national registries on cancer patients in the United States, is intended to provide generalizable information to improve cancer care delivery. Unfortunately, to our knowledge, retrospective studies looking into reasons for refusing standard treatment modalities for NSCLC are lacking.

**Abstract:**

(1) Background: Disparities in cancer treatment and outcomes have long been well-documented in the medical literature. With the eruption of advances in new treatment modalities, the long-existing disparities are now being further uncovered and brought to the attention of the medical community. While social health determinants have previously been linked to treatment disparities in lung cancer, we analyzed data from the National Cancer Database to explore sociodemographic and geographic factors related to accepting or declining physician-recommended chemotherapy. Patients diagnosed with metastatic lung cancer between 2004 and 2016 who declined chemotherapy recommended by their physicians were included in this study. Multivariate logistic regression analysis was performed. Cox Regression and Kaplan-Meier analyses were performed to look for survival characteristics. (2) Results: 316,826 patients with Stage IV lung cancer were identified. Factors related to a higher rate of refusal by patients included older age > 70, female sex, low income, lack of insurance coverage, residency in the New England region, and higher comorbidity. Patients living in areas with lower education were less likely to decline chemotherapy. (3) Conclusion: Further understanding of the factors impacting treatment decisions would be essential to improve the efficacy of care delivery in patients with cancer and reduce reversible causes of disparity.

## 1. Introduction

Lung cancer, of different histologic subtypes, is the leading cause of cancer-related deaths in both men and women in the United States (US) [1]. Non-small cell lung cancer (NSCLC) is the most common subtype, accounting for approximately 85% of all new lung cancer diagnoses worldwide [2]. Unfortunately, despite the significant advances that have been made in the prevention, early detection, and management of NSCLC, the incidence of the advanced stages of NSCLC remains high [3]. The 5-year overall survival rate records a steep decrease from 68% in patients with stage IB disease to around 10% in patients with stage IVA–IVB disease [4,5]. Depending on the stage, histology, genetic alterations, and comorbidities, the treatment approaches in NSCLC usually include surgery, radiotherapy, chemotherapy, immunotherapy, and molecularly targeted therapy, either alone or in combination [6]. However, since most patients with NSCLC have advanced disease at diagnosis, chemotherapy is one of the mainstays of management [7].

Patients diagnosed with cancer face complex treatment decisions that could impact their prognosis. Factors that lead patients to select or refuse treatments are complex and varied [8,9]. Beyond the physicians’ recommendation, patients’ selection of therapy may be influenced by health literacy and understanding [10], financial toxicity [11], and other sociodemographic and cultural factors [12,13]. Additionally, fear of unpleasant side effects from cancer treatment could be one of the most motivating factors to find alternative treatment methods. While refusal of life-prolonging treatment is known to affect survival outcomes negatively, this phenomenon has been scarcely explored [14,15,16]. Furthermore, less is known about the associations of specific geographic regions with rates of refusal of potentially life-prolonging treatment.

Disparities in cancer treatment and outcomes have long been well-documented in the medical literature [17]. With the eruption of advances in new treatment modalities, the long-existing disparities are now being further uncovered and brought to the attention of the medical community. Ultimately, we hope to better understand the need for supportive services like education about disease and financial assistance programs that may guide and aid patients’ treatment-related decision-making. In this analysis, we sought to identify sociodemographic, geographic, and disease characteristics related to decision-making for patients with metastatic lung cancer. In addition, we analyzed the factors associated with prognosis in these patients and explored the impact of treatment-related decisions on survival outcomes. 

## 2. Materials and Methods

### 2.1. Patient Data

The National Cancer Database (NCDB) was queried for patients diagnosed with stage IV lung cancer between 2004 and 2016. NCDB is a national cancer registry with data from over 1500 US medical institutions, supported jointly by the Commission on Cancer (CoC) and the American College of Surgeons (ACS). Relevant patient sociodemographic and clinical factors were included in the study. 

The variable ‘RX_SUMM_CHEMO’ in the NCDB records the type of chemotherapy administered as first-course treatment. If chemotherapy was not administered, it records why it was not administered. In addition, code 87 explicitly records the refusal of recommended chemotherapy by the patients themselves or their family members or guardian. 

Patients were classified into two cohorts according to their decision to accept or refuse chemotherapy treatment. Sociodemographic factors such as the educational attainment of each patient were assessed through records showing the percentage of adults in the patient zip code with no high school degree. The patient’s zip code recorded at the time of diagnosis was matched with files derived from the year 2000 US Census data, and measures of educational attainment were categorized as equally proportional quartiles among all US zip codes by the NCDB. The geographic regions were referenced from those used by the NCDB for the variable entitled ‘FACILITY_LOCATION_CD’. This variable uses the US Census Bureau divisions of the reporting facility to categorize patients based on the state where they received treatment. 

### 2.2. Statistical Analysis

Data analysis for this study was conducted using Statistical Package for the Social Sciences (SPSS), version 28.0 (IBM Corp., Armonk, NY, USA). A multivariable logistic regression model was conducted to assess the association between the dependent variable (refusal of recommended chemotherapy) and a set of explanatory variables, which included age (<70, >70), race, ethnicity, median annual income, education, insurance status, facility type and geographic location, area of residence, distance from the facility, Charlson/Deyo combined comorbidity score (CDCC) 0–3, histology of tumor (squamous, adenocarcinoma or NSCLC, NOS), and tumor grade. In addition, the Cochran-Armitage test was performed to evaluate the trend of refusal rate by year. Finally, Cox regression and Kaplan-Meier analyses were performed to compare survival outcomes based on the included variables. *p* values < 0.05 were considered significant for our study.

## 3. Results

A total of n = 316,826 patients with Stage IV lung cancer were included in this analysis. Age, race, years of diagnosis, median income, CDCC, histology, grade, patient education, insurance status, facility type, and the geographic region of the patient were found to be significantly associated with the decision to either accept or decline recommended chemotherapy (*p* < 0.05) (Table 1). 

As seen in Table 1, patients aged 70 years and older showed increased odds of declining chemotherapy compared to the younger subgroup (OR 2.328, 95% CI 2.266–2.391, *p* < 0.0001). Race showed a statistically significant association with the decision to decline chemotherapy. Interestingly, the Black racial group were less likely to refuse chemotherapy than the White racial group (OR 0.897, 95% CI 0.862–0.933, *p* < 0.001). While minority racial groups, such as American Indians and Aleutian or Eskimo classified as “Others”, did not show a statistically significant difference in the analysis (*p* = 0.263), Asians had the lowest odds of refusal (OR 0.808, 95% CI 0.742–0.880, *p* < 0.001). Similarly, Hispanic ethnicity exhibited comparable decisions related to chemotherapy compared to racial minorities. Hispanics had a lower likelihood ratio (LR) to refuse chemotherapy compared to non-Hispanics (OR 0.805, 95% CI 0.748–0.866, *p* < 0.001). Furthermore, a proportional increase in median income was reported to be significantly associated with a lower probability of chemotherapy refusal. Patients with a median income greater than or equal to $46,000 were the least likely to refuse chemotherapy compared to those with a median income less than $30,000 (OR 0.781, 95% CI 0.744–0.820, *p* < 0.001 vs. OR 1, reference value). Interestingly, patients residing in areas with lower education had lower odds of refusal of chemotherapy (<14% with no high school degree: OR 0.906, 95% CI 0.864–0.949, *p* < 0.001) compared to patients residing in the areas with education levels of 29% or more (OR 1, reference value). The uninsured group had the highest odds of refusal of chemotherapy (OR 1, reference value) followed by those on Medicaid (OR 0.831, 95% CI 0.775–0.890, *p* < 0.001), and ‘other government insurance’ (OR 0.816, 95% CI 0.728–0.913, *p* < 0.001). Additionally, increasing the distance between the area of residence and the treatment center was significantly associated with a decreased likelihood of refusal. Patients residing in areas greater than 25 miles away from the hospital had the lowest rates of refusal of chemotherapy treatment (OR 0.778, 95% CI 0.748–0.809, *p* < 0.001) compared to patients residing in areas less than 5 miles (OR 1, reference value). Patients with a CDCC scoring of 2 and 3 or more were more likely to decline chemotherapy compared to those with no comorbidities on record (OR 1.677, 95% CI 1.615–1.741, *p* < 0.001 and OR 2.046, 95% CI 1.942–2.156, *p* < 0.001, respectively). Lastly, patients with adenocarcinoma histology (OR 0.778, 95% CI 0.755–0.801, *p* < 0.001) and NSCLC NOS (primarily including large cell NSCLC) (OR 0.899, 95% CI 0.869–0.931, *p* < 0.001) were less likely to refuse chemotherapy as compared to those with tumors of squamous cell histology (OR 1, reference value). 

**Figure 1:**
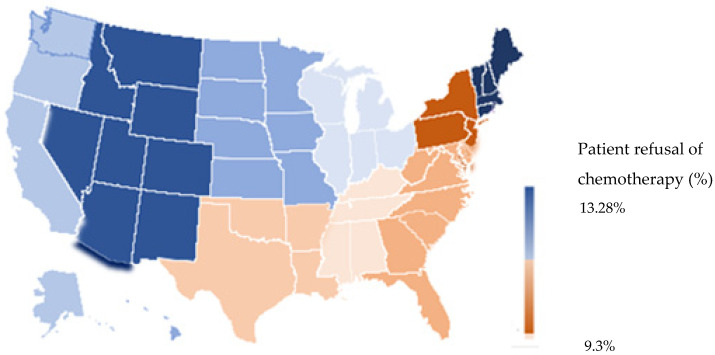
Patient distribution according to geographic location.

Figure 1 portrays the percentage of patients who refused chemotherapy based on the sample of patients in their respective geographic locations. The highest odds of refusal were found in patients from the New England region (OR 1 reference value), followed by the Pacific region (OR 0.942, 95% CI 0.890–0.997, *p* 0.008), followed by the West North Central region (OR 0.910, 95% CI 0.858–0.964, *p* < 0.001), Mountain region (OR 0.901, 95% CI 0.837–0.970, *p* < 0.001), East North Central region (OR 0.834, 95% CI 0.794–0.877, *p* < 0.001), East South Central (OR 0.711, 95% CI 0.669–0.755, *p* < 0.001), West South Central region (OR 0.706, 95% CI 0.663–0.753, *p* < 0.001), South Atlantic region (OR 0.702, 95% CI 0.667–0.739, *p*-value < 0.001), and Middle Atlantic region (OR 0.693, 95% CI 0.657–0.732, *p* < 0.001). Supplementary Table S1 lists and depicts the regional distribution of the states used by the NCDB in its database.

**Figure 2:**
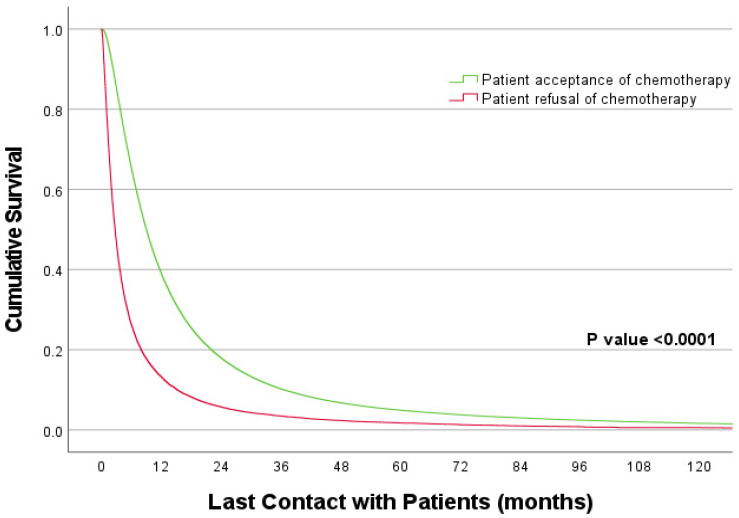
Kaplan Meier curves comparing survival characteristics based on patient decision.

In terms of survival characteristics (Table 2 and Figure 2), refusal of recommended chemotherapy was associated with a significantly poor prognosis (HR 2.312, 95% CI 2.284–2.341, *p* < 0.0001). Demographically, older individuals (HR 1.081, 95% CI 1.071–1.092, *p* < 0.001) and non-Hispanics (HR 1, reference value) were associated with a poor prognosis compared to their younger counterparts (HR 1, reference value) and Hispanics (HR 0.828, 95% CI 0.808–0.849, *p* < 0.001) respectively. In contrast, Asians (HR 0.725, 95% CI 0.705–0.746, *p* < 0.001), patients residing in areas with lower education (<14%: HR 0.972, 95% CI 0.957–0.988, *p* < 0.001), privately insured (HR 0.867, 95% CI 0.849–0.884, *p* < 0.001) and those who received treatment at academic cancer programs (HR 0.884, 95% CI 0.872–0.897, *p* < 0.001) had better prognosis as compared to the rest. In terms of disease characteristics, patients with squamous histology (HR 1, reference value) and undifferentiated tumors (HR 1.538, 95% CI 1.479–1.600, *p* < 0.001) were seen to have lower survival than adenocarcinoma histology (HR 0.912, 95% CI 0.903–0.922) and well-differentiated tumors (HR 1, reference value), respectively. Moreover, the median overall survival (OS) of patients accepting and receiving chemotherapy (Supplementary Table S2, Figure 2) was significantly higher than those refusing this treatment (9.030 months, 95% CI [8.985–9.075] versus 2.730 months, 95% CI [2.685–2.775], log-rank *p* < 0.0001).

Geographically, patients receiving treatment in the West North Central region (HR 1.070, 95% CI 1.048–1.093, *p* < 0.001) were seen to have the worst prognosis, followed by East South Central region (HR 1.037, 95% CI 1.016–1.060, *p* < 0.001), East North Central region (HR 1.024, 95% CI 1.006–1.043, *p* 0.010), West South Central region (HR 0.963, 95% CI 0.942–0.985, *p* 0.001), Mountain states (HR 0.978, CI 0.952–1.004, *p* 0.103) and Middle Atlantic states (HR 0.937, 95% CI 0.920–0.955, *p* < 0.001).

As seen in Supplementary Table S3, there was a gradual increase in the refusal of chemotherapy over time. In addition, the Cochran-Armitage test revealed that this trend by year was statistically significant (*p* < 0.0001).

## 4. Discussion

The complexity of cancer care requires a physician’s clinical reasoning to guide patient treatment and to comprehend patient-related sociodemographic, clinical, and other personal factors influencing treatment judgment. The findings of our analysis are significant as they highlight several sociodemographic, geographic, and disease characteristics influencing patients’ decision-making. Moreover, it emphasizes the considerable reduction in survival for patients who refuse physician-recommended chemotherapy as a part of their cancer therapy.

In our study, elderly patients (>70 years) declined chemotherapy more frequently than the younger subgroup. This was consistent with previous research proving age to be a deterrent to accepting cancer-directed treatment [18]. Our findings are also replicable with a recent study from another national registry that revealed that patients with Stage IV NSCL aged 65 years or older were most likely to be untreated compared with those younger than 65 years old (38.3% versus 22.8%) [19]. As reported in another study, these findings could be related to concerns about the tolerability of chemotherapy, treatment duration, and higher rates of adverse effects of cancer therapy that would be expected in older age groups [20]. Moreover, hearing, visual, and cognitive impairment that impacts the decision-making process in the elderly population may also be significant factors [21]. Racial disparities in refusal of chemotherapy were observed as well. In our analysis, Black and Asian minority racial groups were seen to have higher acceptance of recommended chemotherapy and a better prognosis than others. Moreover, the analysis revealed a significant association of Hispanic ethnicity with decreased LR for patient refusal of therapy, translating into superior survival outcomes compared to non-Hispanics. These findings are in congruence with the results of previous studies that have reported the Hispanic ethnicity to have lower odds of refusing both radiotherapy and chemotherapy for NSCLC [22]. While our research aimed to highlight significant disadvantages of minority racial groups in term of their decisions with respect to chemotherapy administration, the reported findings prove that the white racial group are less likely to adhere to physicians’ recommendation. Plus, it has corroborated the impact of decision-making on clinical outcomes regardless of other sociodemographic and financial advantages. Nonetheless, improving access and addressing economic, sociodemographic, and language barriers to treatment remain priorities to decrease treatment disparities, especially among minorities. Further research to explore a possible variation in perceptions towards the healthcare system by racial and ethnic minorities would help improve the understanding of patient decision-making across diverse cultural contexts, thus increasing overall compliance.

Education status was a significant factor associated with the refusal of recommended chemotherapy, similar to a previous study on patients with lung cancer [23]. In our analysis, patients residing in areas with higher education were seen to have higher rates of refusal of recommended chemotherapy than the rest. These findings deviate from the expected rationale of higher educated patients adhering to physician recommendations and with findings in the literature, whereby patients with lower education had a higher likelihood of refusing cancer treatment [22]. In an attempt to interpret our findings, we hypothesize that patients with higher levels of education likely have better access to information regarding chemotoxicity and cancer treatment options and might rule in favor of treatments other than chemotherapy, or lack thereof, for themselves. This hypothesis may also be supported by our results which demonstrate that patients diagnosed in more recent years were also more likely to refuse treatment. It may seem intuitive that increased familiarity with such treatment modalities would improve adherence to physicians’ recommendations; however, our results reveal an opposing interpretation. Moreover, there might be an intrinsic bias in physicians when interacting with patients with higher levels of education to assume that they understand the pros and cons of chemotherapy over other types of treatment, when in fact, that might not be the case. This might lead to inadequate patient education. In conclusion, these findings point toward the need to inform the patients about the potential risks of refusing lifesaving treatment and the benefits of opting for chemotherapy over other treatments or no treatment, which could help alleviate fears and assist individuals in making informed decisions. 

Another significant factor reported in treatment decision–making is the financial burden associated with cancer treatment [24]. Our data confirmed that the uninsured, followed by those on Medicaid, were found to have a higher association with a refusal of treatment recommended by their treating physicians. Other studies have also reported similar findings [25,26]. Understandably, patients who were either uninsured or on Medicaid were seen to have lower survival in our study. Our analysis also revealed that lower-income patients are more likely to refuse chemotherapy. Financial hardship poses a barrier for many patients with cancer [24,27,28]. It has been reported that patients with cancer commonly use their savings or borrow money to afford cancer treatment [29]. Furthermore, the uninsured are more likely to be diagnosed with advanced-stage disease than the rest [26]. The American Society for Clinical Oncology has raised concerns about inequalities in cancer care due to insurance status [30]. It has been estimated that a lack of financial resources may impact up to two-thirds of patients with cancer [31] and could be associated with more significant anxiety, depression, and suicidal ideation [32]. The financial strain for patients on cancer treatment would be another critical area of emphasis to improve adherence and compliance with cancer therapies. Providing patients with resources, including access to social workers and financial navigators, could enhance the rate of adherence to treatment by educating them about the available opportunities and help reduce the financial burden associated with cancer treatment.

Facility type and location demonstrated significant association with treatment decisions as well. Patients treated at Academic Cancer Programs were seen to have the highest rates of acceptance of recommended chemotherapy and better prognosis compared to the other facility types. We believe the facility-specific approach and counseling could play a role in these findings. Additionally, the highest refusal rates were found in the New England region, followed by the Pacific region and the West North Central region of the US. A previous NCDB-based study on stage III and stage IV patients with breast cancer also reported comparable findings, with the New England region having the highest likelihood of patient refusal of recommended chemotherapy [13]. While the NCDB does not document the specific causes for refusal, several reasons could be behind these observations. These include preconceived notions about chemotherapy and cancers in some areas of the country [33,34] and region-specific limited access to chemotherapy. Higher education rates in the New England region may also be hypothesized to be one of the many factors responsible for the noted lower acceptance rates of recommended chemotherapy, which parallels our findings relating to education and refusal of chemotherapy [35]. Treatment discussions can be overwhelming for patients as cancer management is complex and requires open communication channels between the physician and the patients to guide them toward the appropriate treatment regimen. Future research focusing on studying physicians’ delivery of treatment recommendations and variations in potential communication styles between geographic areas and facility types would be of interest in identifying potential opportunities. Moreover, fears and misconceptions could vary with geographic areas, which might have played a role in receiving therapy or patient hesitancy towards recommended treatment [36,37]. 

Our study had limitations, including the lack of information in the NCDB about the reasons behind the refusal of chemotherapy. As a retrospective study, unmeasured confounders influencing chemotherapy refusal, including patient beliefs and trust in the health care system, could have biased the analysis and affected the results. Furthermore, studies using large databases may be subject to misclassification errors, especially racial/ethnic characterization. Additionally, the NCDB only provides data regarding first-line treatment; patients may have refused therapy at the reporting facility but later received treatment at another center. Another unmeasured bias that may have influenced our analysis is the lack of detailed information on patient comorbidity. Our analysis proves that patients with a higher comorbidity score were more likely to refuse chemotherapy. A more accurate knowledge of the specific comorbidities might have given explanations for refusal, which could be a fear of the progression of underlying disease or heightened anxiety about adverse events. 

Although immunotherapy and precision therapeutics are now routinely used in NSCLC, chemotherapy is still a mainstay of treatment in patients with advanced disease [7]; therefore, the increasing overall trend of chemotherapy refusal noted in our research is worrisome. In addition, with modern communication and social media platforms, patients are increasingly swayed by the misinformation available over the internet [38]. Finally, as noted in other cancers, patients may be lured toward alternative medicine approaches with unproven benefits [39].

## 5. Conclusions

As reported in our study, several sociodemographic factors (including older age, Caucasian race, higher education, lower income, being uninsured, and residence in the New England region) and disease characteristics (including a higher number of comorbidities and squamous histology) were found to be associated with refusal of physician recommended chemotherapy. 

Several factors, including the geographic locations of the patients, were noted to influence adherence to recommended chemotherapy in this study. Understanding the complexity of patient decision-making is vital to successfully delivering cancer treatment. Providing adequate information on the disease, guidance throughout the care process, and identifying patients at risk for refusing treatment is needed. Communication is crucial, and so is establishing trust with patients, gathering information, and assisting patients in shared decisions about care. The quality of communication in cancer care has been shown to affect the patient experience, decision-making, and compliance. Patients who initially refuse treatment may later choose to undergo cancer treatment if given adequate support, information, and time necessary to decide. A patient-centric rather than a disease-centric approach to treatment incorporating patients’ preferences, concerns, and values could help improve compliance. 

Open channels of communication between the treating team and the patients and their loved ones can help patients make informed treatment decisions, as patients with newly diagnosed cancer often hold inaccurate perceptions of their prognosis and treatment expectations [40,41]. A realistic understanding of the goals of cancer therapy could also help patients make better treatment decisions [42,43,44]. Better communication would alleviate the commonly cited barriers to treatment, such as fear of adverse side effects of chemotherapy, uncertainty about treatment effectiveness, and issues surrounding patient-physician relationships and sway decisions against receiving therapy. Patients would be more likely to accept conventional treatment when they feel the healthcare staff have acknowledged and addressed their fears, educated them about treatment possibilities, and allowed them time to adjust to their diagnosis and assimilate information before starting treatment. Furthermore, informing patients of available social services and comprehensive, low-cost insurance programs the government provides is essential to provide optimal care to underprivileged populations. Future research on geographic variation and related factors influencing treatment decisions, as well as innovative methods to tackle those barriers, would be desirable.

## Figures and Tables

**Table 1:** Multivariate logistic regression analysis of factors associated with the decision to decline chemotherapy in patients with metastatic lung cancer.

	Refusal of Chemotherapy		*p*-Value	Multivariate Logistic Regression	*p*-Value
	No N (%)	Yes N (%)		Odds Ratio (95% CI)	
Age (years)			<0.0001		
≤70	188,430 (67.0)	15,176 (42.7)		1	-
>70	92,881 (33.0)	20,339 (57.3)		2.328 (2.266–2.391)	<0.0001
Sex					
Male	153,455 (54.5)	18,488 (52.1)		1	**-**
Female	127,856 (45.5)	17,027 (47.9)		1.164 (1.138–1.191)	<0.001
Race			<0.0001		
White	235,976 (83.9)	30,460 (85.8)		1	-
Black	33,085 (11.8)	3691 (10.4)		0.897 (0.862–0.933)	<0.001
Asian	6496 (2.3)	657 (1.8)		0.808 (0.742–0.880)	<0.001
Others	5754 (2.0)	707 (2.0)		1.048 (0.965–1.137)	0.263
Ethnicity			<0.001		
Non-Hispanic	256,834 (91.3)	32,449 (91.4)		1	-
Hispanic	8431 (3.0)	892 (2.5)		0.805 (0.748–0.866)	<0.001
Others	16,046 (5.7)	2174 (6.1)		1.114 (1.061–1.170)	<0.001
Years of Diagnosis			<0.001		
2004–2007	64,954 (23.1)	6985 (19.7)		1	-
2008–2011	89,065 (31.7)	10,698 (30.1)		1.112 (1.076–1.149)	<0.001
2012–2015	102,595 (36.5)	14,219 (40.0)		1.291 (1.251–1.333)	<0.001
2016–2017	24,697 (8.8)	3613 (10.2)		1.350 (1.291–1.412)	<0.001
Median Income			<0.001		
<$30,000	40,154 (14.3)	5671 (16.0)		1	-
$30,000–$34,999	52,940 (18.8)	7349 (20.7)		0.934 (0.897–0.973)	0.001
$35,000–$45,999	80,814 (28.7)	10,538 (29.7)		0.874 (0.837–0.912)	<0.001
≥$46,000	107,403 (38.2)	11,957 (33.7)		0.781 (0.744–0.820)	<0.001
Education			<0.001		
29% or more	49,226 (17.5)	6514 (18.3)		1	-
20–28.9%	70,074 (24.9)	9251 (26.0)		0.996 (0.959–1.035)	0.839
14–19.9%	69,531 (24.7)	8937 (25.2)		0.958 (0.918–1.000)	0.047
<14%	92,480 (32.9)	10,813 (30.4)		0.906 (0.864–0.949)	<0.001
Insurance status			<0.0001		
Uninsured	11,255 (4.0)	1442 (4.1)		1	-
Private	97,795 (34.8)	6194 (17.4)		0.455 (0.428–0.484)	<0.001
Medicaid	21,499 (7.6)	2574 (7.2)		0.831 (0.775–0.890)	<0.001
Medicare	142,635 (50.7)	24,278 (68.4)		0.718 (0.676–0.763)	<0.001
Other Government	3466 (1.2)	472 (1.3)		0.816 (0.728–0.913)	<0.001
Unknown	4661 (1.7)	555 (1.6)		0.759 (0.682–0.843)	<0.001
Facility type			<0.001		
Community CP *	31,448 (11.2)	4397 (12.3)		1	-
Comprehensive Community CP *	122,998 (43.7)	17,425 (49.1)		1.084 (1.044–1.125)	<0.001
Academic CP *	88,590 (31.5)	8487 (23.9)		0.837 (0.802–0.872)	<0.001
Integrated Network CP *	38,275 (13.6)	5206 (14.7)		1.072 (1.025–1.122)	0.002
Facility location			<0.0001		<0.001
New England	16,611 (5.9)	2545 (7.2)		1	-
Middle Atlantic	43,659 (15.5)	4491 (12.6)		0.693 (0.657–0.732)	<0.001
South Atlantic	61,448 (21.8)	7064 (19.9)		0.702 (0.667–0.739)	<0.001
East North Central	57,207 (20.3)	7678 (21.6)		0.834 (0.794–0.877)	<0.001
East South Central	23,246 (8.3)	2912 (8.2)		0.711 (0.669–0.755)	<0.001
West North Central	24,079 (8.6)	3472 (9.8)		0.910 (0.858–0.964)	<0.001
West South Central	19,583 (7.0)	2235 (6.3)		0.706 (0.663–0.753)	<0.001
Mountain	9197 (3.3)	1336 (3.8)		0.901 (0.837–0.970)	<0.001
Pacific	26,281 (9.3)	3782 (10.6)		0.942 (0.890–0.997)	0.008
Patient Residence			<0.001		
Metro	227,510 (80.9)	28,106 (79.1)		1	-
Urban	42,018 (14.9)	5825 (16.4)		1.072 (1.032–1.115)	<0.001
Rural	6022 (2.1)	858 (2.4)		1.067 (0.983–1.157)	0.120
Unknown	5761 (2.0)	726 (2.0)		0.939 (0.866–1.018)	0.127
Distance (miles)					
<5	89,901 (32.0)	13,233 (37.3)		1	-
5 < distance < 10	61,768 (22.0)	7612 (21.4)		0.900 (0.873–0.929)	<0.001
10 < distance < 25	71,736 (25.5)	8086 (22.8)		0.845 (0.819–0.872)	<0.001
>25	57,906 (20.6)	6584 (18.5)		0.778 (0.748–0.809)	<0.001
Charlson/Deyo score			<0.0001		
0	186,858 (66.4)	19,203 (54.1)		1	-
1	66,112 (23.5)	10,169 (28.6)		1.369 (1.334–1.406)	<0.001
2	20,258 (7.2)	4095 (11.5)		1.677 (1.615–1.741)	<0.001
>3	8083 (2.9)	2048 (5.8)		2.046 (1.942–2.156)	<0.001
Histology			<0.001		
Squamous	50,129 (17.8)	8026 (22.6)		1	-
Adenocarcinoma	162,924 (57.9)	18,723 (52.7)		0.778 (0.755–0.801)	<0.001
NOS	68,258 (24.3)	8766 (24.7)		0.899 (0.869–0.931)	<0.001
Grade			<0.001		
Well-differentiated	6793 (2.4)	789 (2.2)		1	-
Moderately differentiated	29,514 (10.5)	3530 (9.9)		1.036 (0.953–1.126)	0.406
Poorly differentiated	77,956 (27.7)	9190 (25.9)		1.085 (1.002–1.174)	0.044
Undifferentiated	4503 (1.6)	532 (1.5)		1.068 (0.947–1.205)	0.282
Unknown	162,545 (57.8)	21,474 (60.5)		1.232 (1.141–1.331)	<0.001

* CP Cancer Program.

**Table 2:** Survival characteristics of patients with metastatic non-small cell lung cancer.

	Hazard Ratio (95% CI)	*p*-Value
Patient’s decision on recommended chemotherapy		
Acceptance of treatment	1	-
Refusal of treatment	2.312 (2.284–2.341)	<0.0001
Age (years)		
≤70	1	-
>70	1.081 (1.071–1.092)	<0.001
Race		
White	1	<0.001
Black	0.922 (0.910–0.934)	<0.001
Asian	0.725 (0.705–0.746)	<0.001
Others	0.854 (0.830–0.879)	<0.001
Ethnicity		
Non-Hispanic	1	-
Hispanic	0.828 (0.808–0.849)	<0.001
Others	1.043 (1.027–1.060)	<0.001
Years of Diagnosis		
2004–2007	1	-
2008–2011	0.945 (0.936–0.955)	<0.001
2012–2015	0.862 (0.853–0.870)	<0.001
Median Income		
<$30,000	1	-
$30,000–$34,999	0.977 (0.963–0.991)	0.001
$35,000–$45,999	0.965 (0.951–0.979)	<0.001
≥$46,000	0.968 (0.952–0.983)	<0.001
Education		
29% or more	1	-
20–28.9%	1.010 (0.997–1.022)	0.133
14–19.9%	0.998 (0.984–1.012)	0.777
<14%	0.972 (0.957–0.988)	<0.001
Insurance status		
Uninsured	1	-
Private insurance	0.867 (0.849–0.884)	<0.001
Medicaid	0.968 (0.945–0.991)	<0.001
Medicare	0.943 (0.924–0.963)	<0.001
Other Government	0.937 (0.900–0.975)	0.001
Unknown	0.982 (0.948–1.017)	0.302
Facility type		
Community CP *	1	-
Comprehensive Community CP *	0.987 (0.975–1.000)	0.055
Academic CP *	0.884 (0.872–0.897)	<0.001
Integrated Network CP *	0.968 (0.953–0.983)	<0.001
Facility location		
New England	1	-
Middle Atlantic	0.937 (0.920–0.955)	<0.001
South Atlantic	1.013 (0.995–1.031)	0.163
East North Central	1.024 (1.006–1.043)	0.010
East South Central	1.037 (1.016–1.060)	<0.001
West North Central	1.070 (1.048–1.093)	<0.001
West South Central	0.963 (0.942–0.985)	0.001
Mountain	0.978 (0.952–1.004)	0.103
Pacific	1.004 (0.984–1.024)	0.715
Patient Residence		
Metro	1	
Urban	1.030 (1.017–1.044)	<0.001
Rural	1.029 (1.00–1.058)	0.051
Unknown	1.013 (0.986–1.042)	0.346
Distance (miles)		
<5	1	-
5 < distance < 10	0.995 (0.984–1.005)	0.326
10 < distance < 25	0.977 (0.967–0.988)	<0.001
>25	0.925 (0.913–0.937)	<0.001
Other	1.024 (0.627–1.672)	0.925
Charlson/Deyo score		
0	1	-
1	1.136 (1.125–1.146)	<0.001
2	1.223 (1.205–1.241)	<0.001
>3	1.343 (1.313–1.374)	<0.001
Histology		
Squamous	1	-
Adenocarcinoma	0.912 (0.903–0.922)	<0.001
NOS	1.060 (1.048–1.073)	<0.001
Grade		
Well-differentiated	1	-
Moderately differentiated	1.195 (1.162–1.229)	<0.001
Poorly differentiated	1.485 (1.446–1.525)	<0.001
Undifferentiated	1.538 (1.479–1.600)	<0.001
Unknown	1.414 (1.378–1.451)	<0.001

* CP Cancer Program.

## Data Availability

All data used during this study are available online at https://www.facs.org/qualityprograms/cancer/ from the National Cancer Database, accessed on 4 February 2023.

## Supplementary Materials

The following supporting information can be downloaded at: https://www.mdpi.com/article/10.3390/cancers15061686/s1, Table S1: US Census division of the states used by the NCDB; Table S2: Median overall survival based on the patient’s decision to accept or refuse recommended chemotherapy; Table S3: Change in the trend of refusal of recommended chemotherapy from 2004–2016.
