# Supplementary material for: Factors Associated with the Decision to Decline Chemotherapy in Metastatic Non-Small Cell Lung Cancer
Source: Cancers (Basel). 2023 Mar 9;15(6):1686. doi: 10.3390/cancers15061686 (PMC10046757; doi:10.3390/cancers15061686)
Supplement: Supplementary file 1 [file cancers-15-01686-s001.zip › cancers-2225685-supplementary.pdf]

**Supplementary Table S1.** US Census division of the states used by the NCDB

| REGION | STATES INCLUDED |
|---|---|
| **New England** | CT, MA, ME, NH, RI, VT |
| **Middle Atlantic** | NJ, NY, PA |
| **South Atlantic** | DC, DE, FL, GA, MD, NC, SC, VA, WV |
| **East North Central** | IL, IN, MI, OH, WI |
| **East South Central** | Al, KY, MS, TN |
| **West North Central** | IA, KS, MN, MO, ND, NE, SD |
| **West South Central** | AR, LA, OK, TX |
| **Mountain** | AZ, CO, ID, MT, NM, NV, UT, WY |
| **Pacific** | AK, CA, HI, OR, WA |

**Supplementary Table S2.** Median overall survival based on the patient's decision to accept or refuse recommended chemotherapy

| | **Estimate (months)** | **Std. Error** | **95% Confidence Interval** |
|---|---|---|---|
| Acceptance of recommended treatment | 9.030 | 0.023 | 8.985-9.075 |
| Refusal of recommended treatment | 2.730 | 0.023 | 2.685-2.775 |
| Overall | 8.180 | 0.021 | 8.139-8.221 |
| * Significant (p<0.0001) on both Log-rank and Breslow test | | | |

**Supplementary Table S3.** Change in the trend of refusal of recommended chemotherapy from 2004 – 2016

| | **Chemotherapy Refusal (N)** | **Chemotherapy Acceptance (N)** | **Refusal rate (%)** |
|---|---|---|---|
| 2004 | 1643 | 15,045 | 9.8 |
| 2005 | 1648 | 15,923 | 9.4 |
| 2006 | 1798 | 16,496 | 9.8 |
| 2007 | 1896 | 17,490 | 9.8 |
| 2008 | 2255 | 20,047 | 10.1 |
| 2009 | 2415 | 21,462 | 10.1 |
| 2010 | 3025 | 23,595 | 11.4 |
| 2011 | 3003 | 23,961 | 11.1 |
| 2012 | 3314 | 24,597 | 11.9 |
| 2013 | 3508 | 25,867 | 11.9 |
| 2014 | 3671 | 26,829 | 12.3 |
| 2015 | 3726 | 25,842 | 12.6 |
| 2016 | 3613 | 24,697 | 12.8 |
| Total | 35,515 | 281,311 | |

The Cochran-Armitage test revealed that the refusal rate increased by year ( $p$ <0.001).
